# Supplementary material for: Abiotic Treatment to Common Bean Plants Results in an Altered Endophytic Seed Microbiome
Source: Microbiol Spectr. 2022 Apr 4;10(2):e00210-21. doi: 10.1128/spectrum.00210-21 (PMC9045313; doi:10.1128/spectrum.00210-21)
Supplement: SUPPLEMENTAL FILE 1 — Supplemental material. Download SPECTRUM00210-21_Supp_1_seq5.pdf, PDF file, 0.5 MB [file spectrum00210-21_supp_1_seq5.pdf]

Supplementary Information accompanying

Abiotic treatment to common bean plants results in an altered endophytic seed microbiome

by

A. Fina Bintarti, Patrick J. Kearns, Abby Sulesky-Grieb, and Ashley Shade

Supplementary Tables

Table S1: One-half strength Hoagland Solution recipe provided by the Michigan State University growth chamber facility.

Nutrient	Grams of nutrient per 40L solution
**Solution A***	
Calcium nitrate	11800.00
Sequestrene	1537.60
**Solution B***	
Potassium phosphate, monobasic	1370.00
Potassium nitrate	5066.00
Magnesium sulphate	2500.00
Zinc sulphate	2.24
Manganous sulphate	15.64
Copper sulphate	0.84
Boric acid	29.00
Molybdic acid	0.20
*Both solution A and B are used at 1:500 ratios (76.9 ppm)	
KOH is added to adjust the final nutrient solution pH	

Table S2: Soil pH, organic matter content (OM) and nutrient content for untreated bulk soil and post-treatment soil collected after plant harvest.

Sample	Treatment	pH	P (ppm)	K (ppm)	Ca (ppm)	Mg (ppm)	OM (%)	NO3 (ppm)	NH4 (ppm)
**bulk 1**	none	7.3	32	39	1506	154	3.1	25.6	7.1
**bulk 2**	none	7.3	35	39	1627	170	3.2	25.9	7.6
**bulk 3**	none	7.3	38	39	1673	174	3.1	24.6	6.4
**n7**	nitrogen	7.7	36	94	1659	187	3.4	28.3	0.7
**n6**	nitrogen	7.4	73	96	1532	161	3.8	24.8	0.7
**n5**	nitrogen	7.6	86	153	1587	162	4.8	21.8	0.4
**n4**	nitrogen	7.4	69	97	1527	157	3.4	26.7	0.9
**n3**	nitrogen	7.6	69	141	1533	149	2.9	19.0	1.4
**n2**	nitrogen	7.6	63	162	1509	156	3.2	29.6	1.5
**n1**	nitrogen	7.7	46	104	1651	201	3.9	20.7	1.7
**n8**	nitrogen	7.7	42	46	1679	178	3.2	11.5	0.1
**d1**	drought	7.9	32	39	1638	172	3.1	3.0	0.7
**d2**	drought	7.7	34	41	1644	184	3.3	37.3	1.4
**d3**	drought	7.7	31	39	1599	176	4.2	2.9	1.3
**d4**	drought	7.8	26	39	1547	160	3.2	3.8	0.8
**d5**	drought	7.6	28	39	1636	176	3.4	2.5	0.8
**d6**	drought	7.8	31	39	1649	189	3.3	21.2	1.1
**d7**	drought	7.7	30	39	1752	187	4.1	3.4	1.0
**d8**	drought	7.8	34	43	1588	176	3.0	42.4	1.4
**c1**	control	7.7	29	39	1648	158	3.2	0.6	1.4
**c2**	control	8.0	30	39	1607	164	3.1	6.7	1.2
**c3**	control	7.9	34	38	1657	135	3.4	0.5	1.0
**c4**	control	7.9	27	38	1574	129	3.3	0.5	1.2
**c5**	control	7.9	32	38	1699	163	3.7	0.9	0.7
**c6**	control	7.9	32	38	1567	151	3.6	0.3	0.6
**c7**	control	7.8	30	38	1669	157	3.6	0.8	1.4
**c8**	control	7.9	26	39	1747	219	4.9	2.4	1.1

Table S3: Comparison of plant biomass among treatments. Bolded rows are significantly different as determined by Kruskal-Wallis test and post-hoc Dunn's test with Benjamini-Hochberg false discovery rate (FDR) correction.

Kruskal-Wallis test				Post-hoc Dunn's test with Benjamini-Hochberg FDR correction			
**Parameter**	**Chi-squared**	**df**	**p-val**	**Comparison**	**Z**	**P. unadj**	**P. adj**
**Shoot mass**	20.165	2	**0.00004**	Control – Nutrient addition	-2.29810	0.02156	**0.03233**
				Control – Water withholding	2.19203	0.02838	**0.02838**
				Nutrient addition – Water withholding	4.49013	0.00001	**0.00002**
**Root mass**	15.365	2	**0.00046**	Control – Nutrient addition	-3.42947	0.00060	**0.00181**
				Control – Water withholding	-0.07071	0.94363	0.94363
				Nutrient addition – Water withholding	3.35876	0.00078	**0.00117**
**Pod mass**	18.305	2	**0.00010**	Control – Nutrient addition	-2.08597	0.03698	**0.03698**
				Control –Water withholding	2.19203	0.02838	**0.04257**
				Nutrient addition – Water withholding	4.27800	0.00002	**0.00006**
**Pod number**	17.973	2	**0.00013**	Control – Nutrient addition	-2.53347	0.01129	**0.01694**
				Control – Water withholding	1.67708	0.09353	0.09353
				Nutrient addition – Water withholding	4.21055	0.00003	**0.00008**

Table S4: Comparison of rhizosphere soil chemistry of the three treatments using one-way ANOVA or Kruskal-Wallis test. Bolded rows are significantly different as determined by Kruskal-Wallis test and post-hoc Dunn’s test with Benjamini-Hochberg false discovery rate (FDR) correction. Post-hoc tests were not performed for samples with insignificant p-values from Kruskal-Wallis or ANOVA tests.

	Kruskal-Wallis test			Post-hoc Dunn’s test with Benjamini-Hochberg FDR correction			
**Parameter**	**Chi-squared**	**df**	**p-value**	**Comparison**	**Z**	**P. unadj**	**P. adj**
pH	14.634	2	**0.00066**	Control – Nutrient addition	3.824263	0.00013	**0.00039**
				Control – Water withholding	1.83057	0.06716	0.06716
				Nutrient addition – Water withholding	-1.99369	0.04619	0.06928
P	15.613	2	**0.00041**	Control – Nutrient addition	-3.60033	0.00032	**0.00095**
				Control – Water withholding	-0.39018	0.69640	0.69640
				Nutrient addition – Water withholding	3.21014	0.00133	**0.00199**
K	19.172	2	**0.00007**	Control – Nutrient addition	-4.34149	0.00001	**0.00004**
				Control –Water withholding	-1.67822	0.09330	0.09330
				Nutrient addition – Water withholding	2.66327	0.00774	**0.01161**
$NO3^-$	14.261	2	**0.0008**	Control – Nutrient addition	-3.67776	0.00024	**0.00071**
				Control –Water withholding	-2.58150	0.00984	**0.01476**
				Nutrient addition – Water withholding	1.09625	0.27297	0.27297
Mg	4.8514	2	0.08842				
OM	0.53985	2	0.7634				
	**One-way ANOVA test**						
**Parameter**	**F-value**	**df**	**p-value**				
Ca	2.035	2	0.156				
$NH4^+$	0.343	2	0.713				

Table S5: Bacterial genera identified in <1% of the OTUs in each seed microbiome sample.

Treatment	Sample	Bacterial Genus
**Control**	C1	Unidentified Steroidobacteraceae
	C2	Rhizobiales
	C4	Solibacillus
	C5	Virgibacillus<br>Brevibacillus<br>Sporosarcina
	C7	Brevibacillus
	C8	Brevibacillus
**Water withholding**	D1	Dyadobacter<br>Anaerobacillus<br>Virgibacillus<br>Paenibacillus<br>Solibacillus
	D2	Anaerobacillus<br>Solibacillus
	D3	Anaerobacillus<br>Bacillaceae<br>Solibacillus<br>Sporosarcina
	D4	Anaerobacillus<br>Bacillaceae<br>Solibacillus
	D5	Anaerobacillus<br>Bacillaceae<br>Solibacillus
	D6	Pedobacter<br>Anaerobacillus<br>Bacillaceae<br>Sporosarcina<br>Escherichia-Shigella
	D7	Dyadobacter<br>Anaerobacillus<br>Bacillaceae<br>Sporosarcina<br>Escherichia-Shigella
	D8	Anaerobacillus<br>Bacillaceae<br>Domibacillus<br>Solibacillus<br>Sporosarcina<br>Escherichia-Shigella<br>Pseudomonas
**Nutrient addition**	N1	Blastococcus<br>Promicromonospora<br>Anaerobacillus<br>Solibacillus
	N2	Pedobacter<br>Anaerobacillus<br>Solibacillus
	N3	Anaerobacillus<br>Solibacillus<br>Nitrospira
	N4	Sphingobacterium<br>Anaerobacillus<br>Bacillaceae<br>Fictibacillus<br>Paenibacillus<br>Sporosarcina<br>Colwelliaceae<br>Escherichia-Shigella
	N5	Sphingobacterium<br>Anaerobacillus<br>Bacillaceae<br>Fictibacillus<br>Paenibacillus<br>Solibacillus<br>Sporosarcina<br>Colwelliaceae<br>Escherichia-Shigella
	N6	Sphingobacterium<br>Anaerobacillus<br>Bacillaceae<br>Fictibacillus<br>Paenibacillus<br>Sporosarcina<br>Colwelliaceae<br>Escherichia-Shigella
	N7	Blastococcus<br>Bacillaceae<br>Virgibacillus
	N8	Virgibacillus<br>Sporosarcina

Table S6: Fungal genera identified in <10% of the OTUs in each seed microbiome sample.
OTUs that could not be identified at the Genus level are identified at the Order or Family level as labeled below.

Treatment	Sample	Fungal Genus
**Control**	C1	Alternaria
		Order: Pleosporales
		Penicillium
		Family: Aspergillaceae
		Family: Nectriaceae
		Order: Hypocreales
		Conocybe
		Fibulochlamys
		Ischnoderma
		Filobasidium
		Mortierella
		Unidentified
	C2	Order: Pleosporales
		Penicillium
		Family: Aspergillaceae
		Peziza
		Meyerozyma
		Verticillium
		Family: Nectriaceae
		Order: Hypocreales
		Conocybe
		Fibulochlamys
		Parasola
		Guehomyces
		Filobasidium
		Wallemia
		Mortierella
		Unidentified
	C3	Penicillium
		Family: Aspergillaceae
		Family: Chaetomiaceae
		Sistotrema
		Guehomyces
		Wallemia
		Unidentified
		Unidentified
	C4	Unidentified
		Penicillium
		Family: Aspergillaceae
		Family: Nectriaceae
		Order: Hypocreales
		Apodus
		Wallemia
		Unidentified
	C5	Alternaria
		Order: Pleosporales
		Penicillium
		Family: Aspergillaceae
		Family: Trichocomaceae
		Meyerozyma
		Fusarium
		Gibberella
		Family: Nectriaceae
**Nutrient Addition**	N1	Alternaria
		Order: Pleosporales
		Unidentified
		Penicillium
		Family: Aspergillaceae
		Family: Pyronemataceae
		Family: Nectriaceae
		Order: Hypocreales
		Unidentified
		Marasmius
		Wallemia
		Olpidium
	N2	Alternaria
		Order: Pleosporales
		Unidentified
		Family: Aspergillaceae
		Emericellopsis
		Gibberella
		Family: Nectriaceae
		Order: Hypocreales
		Unidentified
		Fibulochlamys
		Coprinellus
		Guehomyces
		Wallemia
		Mortierella
	N3	Alternaria
		Order: Pleosporales
		Unidentified
		Family: Aspergillaceae
		Verticillium
		Gibberella
		Family: Nectriaceae
		Order: Hypocreales
		Unidentified
		Guehomyces
		Wallemia
	N4	Alternaria
		Order: Pleosporales
		Unidentified
		Family: Sclerotiniaceae
		Family: Pyronemataceae
		Cyberlindnera
		Fusarium
		Gibberella
		Family: Nectriaceae
		Order: Hypocreales
		Order: Microascales
		Humicola
		Apodus
		Family: Lasiosphaeriaceae
		Unidentified
		Parasola
**Water Withholding**	D1	Alternaria
		Drechslera
		Order: Pleosporales
		Unidentified
		Aspergillus
		Family: Aspergillaceae
		Family: Pyronemataceae
		Verticillium
		Fusarium
		Gibberella
		Family: Nectriaceae
		Order: Hypocreales
		Unidentified
		Conocybe
		Fibulochlamys
		Cyathus
		Order: Auriculariales
		Sistotrema
		Uncobasidium
		Udeniozyma
		Guehomyces
		Unidentified
		Mortierella
		Family: Mortierellaceae
		Olpidium
	D2	Alternaria
		Order: Pleosporales
		Unidentified
		Order: Chaetothyriales
		Aspergillus
		Family: Orbiliaceae
		Gibberella
		Family: Nectriaceae
		Order: Hypocreales
		Order: Sordariales
		Unidentified
		Sistotrema
		Order: Tremellodendropsidales
		Guehomyces
		Unidentified
		Family: Mortierellaceae
		Unidentified
	D3	Alternaria
		Order: Pleosporales
		Unidentified
		Order: Chaetothyriales
		Aspergillus
		Trichoderma
		Gibberella
		Family: Nectriaceae
		Order: Hypocreales
		Order: Microascales
		Apodus

Treatment	Sample	Taxon
**Control**	C5	Order: Hypocreales
		Unidentified
		Schizophyllum
		Order: Tremellales
		Wallemia
		Olpidium
	C6	Alternaria
		Order: Pleosporales
		Unidentified
		Penicillium
		Family: Aspergillaceae
		Oidiodendron
		Verticillium
		Fusarium
		Family: Nectriaceae
		Order: Hypocreales
		Order: Microascales
		Order: Auriculariales
		Guehomyces
		Wallemia
	C7	Alternaria
		Order: Pleosporales
		Penicillium
		Family: Aspergillaceae
		Talaromyces
		Family: Trichocomaceae
		Peziza
		Clonostachys
		Fusarium
		Family: Nectriaceae
		Order: Hypocreales
		Chaetomium
		Humicola
		Zopfiella
		Family: Chaetomiaceae
		Order: Sordariales
		Unidentified
		Family: Ceratobasidiaceae
		Order: Tremellales
		Wallemia
		Spizellomyces
		Claroideoglomus
		Mortierella
		Olpidium
		Unidentified
	C8	Unidentified
		Penicillium
		Family: Aspergillaceae
		Talaromyces
		Pleuroascus
		Peziza
		Fusarium
		Gibberella
		Order: Hypocreales
		Order: Microascales
		Trichocladium
		Family: Lasiosphaeriaceae
		Order: Sordariales
		Unidentified
**Nutrient Addition**	N4	Order: Auriculariales
		Sistotrema
		Hymenochaete
		Ischnoderma
		Order: Tremellodendropsidales
		Udeniozyma
		Guehomyces
		Itersonilia
		Naganishia
		Order: Tremellales
		Wallemia
		Unidentified
		Mortierella
		Family: Mortierellaceae
	N5	Alternaria
		Order: Pleosporales
		Unidentified
		Exophiala
		Order: Chaetothyriales
		Family: Aspergillaceae
		Family: Trichocomaceae
		Glarea
		Order: Helotiales
		Family: Pyronemataceae
		Meyerozyma
		Fusarium
		Gibberella
		Family: Nectriaceae
		Order: Hypocreales
		Humicola
		Unidentified
		Fibulochlamys
		Sistotrema
		Guehomyces
		Naganishia
		Wallemia
		Unidentified
		Unidentified
		Mortierella
	N6	Alternaria
		Order: Pleosporales
		Unidentified
		Order: Helotiales
		Family: Pseudeurotiaceae
		Family: Pyronemataceae
		Cyberlindnera
		Verticillium
		Fusarium
		Gibberella
		Family: Nectriaceae
		Order: Hypocreales
		Chaetomium
		Humicola
		Family: Chaetomiaceae
		Apodus
		Family: Lasiosphaeriaceae
		Unidentified
		Sistotrema
		Guehomyces
**Water Withholding**	D3	Order: Sordariales
		Unidentified
		Marasmius
		Cyathus
		Sistotrema
		Guehomyces
		Order: Tremellales
		Unidentified
		Mortierella
	D4	Alternaria
		Order: Pleosporales
		Family: Aspergillaceae
		Verticillium
		Gibberella
		Family: Nectriaceae
		Order: Hypocreales
		Chaetomium
		Conocybe
		Fibulochlamys
		Cyathus
		Order: Agaricales
		Ischnoderma
		Filobasidium
		Wallemia
		Mortierella
		Unidentified
	D5	Penicillium
		Family: Aspergillaceae
		Chaetomium
		Cercophora
		Guehomyces
		Wallemia
		Unidentified
		Mortierella
		Unidentified
	D6	Alternaria
		Order: Pleosporales
		Unidentified
		Family: Aspergillaceae
		Peziza
		Fusarium
		Family: Nectriaceae
		Order: Hypocreales
		Humicola
		Podospora
		Unidentified
		Conocybe
		Parasola
		Guehomyces
		Wallemia
		Mortierella
	D7	Pyrenochaeta
		Alternaria
		Order: Pleosporales
		Penicillium
		Family: Trichocomaceae
		Oidiodendron
		Unidentified
		Meyerozyma

**Control**	C8	Fibulochlamys	**Nutrient Addition**	N6	Unidentified	**Water Withholding**	D7	Acremonium
		Sistotrema			Wallemia			Fusarium
		Guehomyces			Unidentified			Family: Nectriaceae
		Itersonilia			Ramicandelaber			Order: Hypocreales
		Order: Tremellales			Mortierella			Trichocladium
		Wallemia			Olpidium			Zopfiella
		Claroideoglomus		N7	Alternaria			Family: Chaetomiaceae
		Rhizophagus			Order: Pleosporales			Order: Sordariales
		Order: Paraglomerales			Exophiala			Unidentified
		Mortierella			Penicillium			Unidentified
		Unidentified			Talaromyces			Fibulochlamys
					Botrytis			Guehomyces
					Peziza			Wallemia
					Chaetosphaeria			Spizellomyces
					Clonostachys			Funneliformis
					Trichoderma			Unidentified
					Fusarium			Mortierella
					Gibberella			Olpidium
					Family: Nectriaceae			
					Order: Hypocreales			
					Family: Halosphaeriaceae			
					Zopfiella			
					Family: Chaetomiaceae			
					Order: Sordariales			
					Unidentified			
					Fibulochlamys			
					Order: Agaricales			
					Solicoccozyma			
					Wallemia			
					Spizellomyces			
					Unidentified			
					Claroideoglomus			
					Family: Claroideoglomeraceae			
					Family: Glomeraceae			
					Unidentified			
					Mortierella			

Table S7: Shared bacterial taxa across treatments (occupancy of 1)

OTU.ID	Occ	Domain	Phylum	Class	Order	Family	Genus	Species
**Shared taxa between control and water withholding-treated seed samples**								
EU231617.1.1548	1	Bacteria	Firmicutes	Bacilli	Bacillales	Bacillaceae	*Bacillus*	NA
HQ318731.1.1489	1	Bacteria	Firmicutes	Bacilli	Bacillales	Bacillaceae	*Bacillus*	*B. flexus*
HQ323430.1.1541	1	Bacteria	Firmicutes	Bacilli	Bacillales	Bacillaceae	*Bacillus*	*B. megaterium*
KP232906.1.1565	1	Bacteria	Firmicutes	Bacilli	Bacillales	Bacillaceae	*Bacillus*	NA
**Shared taxa between control and nutrient-treated seed samples**								
EU231617.1.1548	1	Bacteria	Firmicutes	Bacilli	Bacillales	Bacillaceae	*Bacillus*	NA
HQ323430.1.1541	1	Bacteria	Firmicutes	Bacilli	Bacillales	Bacillaceae	*Bacillus*	*B. megaterium*
KP232906.1.1565	1	Bacteria	Firmicutes	Bacilli	Bacillales	Bacillaceae	*Bacillus*	NA

Note: Occ = occupancy; NA = Not Assigned

Table S8: Shared fungal taxa across treatments (occupancy of 1)

OTU.ID	Occ	Domain	Phylum	Class	Order	Family	Genus	Species
**Shared taxa between control and water withholding-treated seed samples**								
OTU_24	1	Fungi	Ascomycota	Eurotiomycetes	Eurotiales	Aspergillaceae	*Aspergillus*	*A. niger*
OTU_248	1	Fungi	NA	NA	NA	NA	NA	NA
OTU_26	1	Fungi	Ascomycota	Eurotiomycetes	Eurotiales	Aspergillaceae	*Aspergillus*	*A. niger*
OTU_27	1	Fungi	Ascomycota	Eurotiomycetes	Eurotiales	Aspergillaceae	*Aspergillus*	NA
**Shared taxa between control and nutrient-treated seed samples**								
OTU_151	1	Fungi	Ascomycota	Eurotiomycetes	Eurotiales	Aspergillaceae	*Penicillium*	*P. astrolabium*
OTU_19	1	Fungi	Ascomycota	Eurotiomycetes	Eurotiales	Aspergillaceae	*Aspergillus*	*A. niger*
OTU_24	1	Fungi	Ascomycota	Eurotiomycetes	Eurotiales	Aspergillaceae	*Aspergillus*	*A. niger*
OTU_248	1	Fungi	NA	NA	NA	NA	NA	NA
OTU_26	1	Fungi	Ascomycota	Eurotiomycetes	Eurotiales	Aspergillaceae	*Aspergillus*	*A. niger*
OTU_27	1	Fungi	Ascomycota	Eurotiomycetes	Eurotiales	Aspergillaceae	*Aspergillus*	NA

Note: Occ = occupancy; NA = Not Assigned

Supplementary Figures

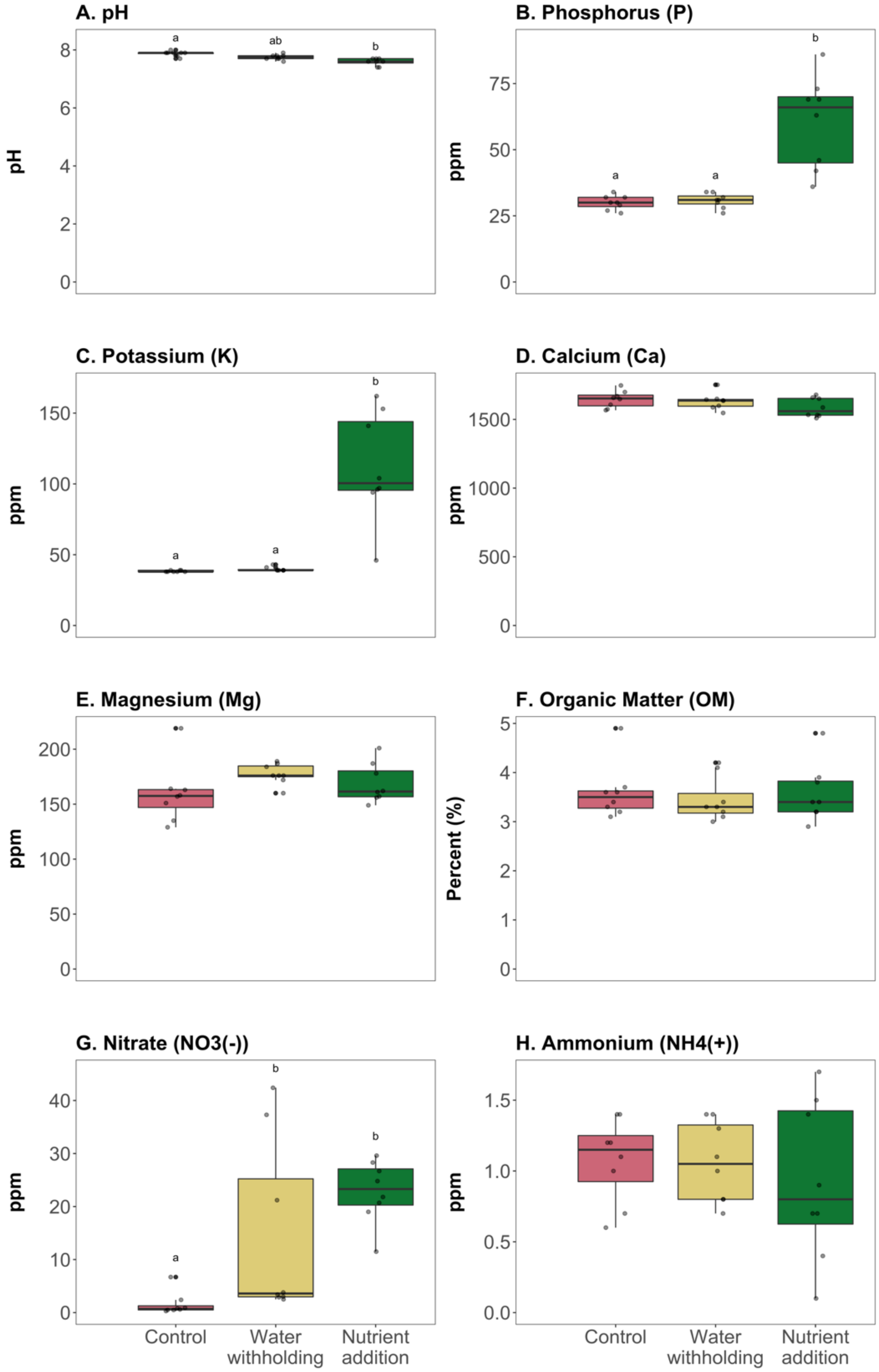

Figure S1: Plant rhizosphere soil chemistry for control, water withholding, and nutrient addition treatments of common bean. For each box plot, circles represent one rhizosphere measurement within a treatment. The central horizontal lines represent the mean of measurement, the outer horizontal lines of the box represent the 25th and 75th percentiles. Boxes labelled with different letters are identified as significantly different by a Kruskal-Wallis and post-hoc Dunn's test with a Benjamini-Hochberg false discovery rate correction (p-value significance ranges from <0.05 to <0.0001).

Supplementary Methods

Soil testing at the Michigan State University Soil Plant and Nutrient Laboratory

Soil samples are dried in a forced air oven at 35°C, then ground with a flail grinder and sieved through a 10 mesh screen.

pH Determination: 5 grams of the dried and sieved soil sample is combined with 10 mL distilled water. The mixture is stirred and allowed to stand for 15 minutes. After 15 minutes the mixture is stirred again and immediately tested with a digital pH meter standardized with pH 4.0 and 7.0 buffers.

Bray P1 Method for Determining Extractable Phosphorous: 2 grams of dried and sieved soil is combined with 20 mL prepared Bray P1 Working Solution A (0.03 N NH_4F – .025 N HCl) and shaken for 5 minutes at 180-200 rpm. Soil particles are then filtered out of the solution using a Whatman #1 filter. 2 mL of filtered soil extract is then combined with 18 mL prepared Working Solution B (Acid Molybdate – Ascorbic Acid solution) and allowed to sit for 15 minutes while color develops. Color is then read on a spectrophotometer at 660 nm and compared to a standard phosphorous curve to determine concentration.

Extractable Potassium, Calcium, and Magnesium: 2.5 grams of prepared soil is combined with 20 mL prepared 20 ml 1N neutral Ammonium Acetate (NH_4OAc) extracting solution and shaken for 5 minutes at 180-200 rpm. Soil particles are then filtered out of the solution using a Whatman #1 filter. Nutrient levels are determined using a colorimetric test in a Technicon Autoanalyzer. For magnesium, Magnesium Blue working solution will react with magnesium in the sample solution to develop a blue color. Intensity of the blue color is an indication of the Mg present. For calcium and potassium, sample solutions are mixed automatically with $LiNO_3$ working solution and fed into a flame photometer. Lithium serves as in internal reference standard for determination of Ca and K.

Organic matter: 1 gram of prepared soil is combined with 10 ml of $Na_2Cr_2O_7$ solution. 10 ml of 96% concentrated sulfuric acid is added and the mixture is allowed to react for 30 minutes. Mixture is then diluted with 15 mL distilled water and allowed to stand overnight. After standing period, 5 mL supernatant is combined with 5 mL distilled water in a colorimeter tube. Development of orange color in sample is read on a colorimeter calibrated by a standard curve for % organic matter.

Nitrate Nitrogen: 10 g dry soil and is combined with 50mL 1 N KCl, shaken for 30 minutes at 180-200 rpm and filtered through a Whatman #2 filter. Nitrate-N content of the filtered soil extract is then determined using either the nitrate-reduction method (QuikChem Method No. 10-107-04-1-A) through a LaChat Rapid Flow Injection Unit, or using a nitrate selective ion electrode connected to an appropriate meter, standardized with prepared nitrate standards.

Total Nitrogen - Micro-Kjeldahl Block Digestion: 0.5 g prepared soil is placed in a 100 mL digestion tube with 1 KC-C5 catalyst tablet (5 g K2 SO4 + 150 mg CuSO4) and 7 mL concentrated sulfuric acid. Sample is mixed carefully and placed in a block digestor for 1 1⁄2 hours at 375° C. Sample is then removed from digestor block, allowed to cool for 20 to 30 minutes, and filled half full with warm distilled water, thoroughly mixing water with sample. The sample is then filtered through a #2 Whatman filter and analyzed with the LaChat Rapid Flow Injection Unit using the ammonia-salicylate method and appropriate standard.
